# Vaccination coverage and reasons for non-vaccination in a district of Istanbul

**DOI:** 10.1186/1471-2458-6-125

**Published:** 2006-05-05

**Authors:** Sebahat D Torun, Nadi Bakırcı

**Affiliations:** 1 Marmara University Medical Faculty Department of Public Health, Istanbul, Turkey; 2 Assistant Professor of Public Health, Marmara University Medical Faculty Department of Public Health, Istanbul, Turkey

## Abstract

**Background:**

In order to control and eliminate the vaccine preventable diseases it is important to know the vaccination coverage and reasons for non-vaccination. The primary objective of this study was to determine the complete vaccination rate; the reasons for non-vaccination and the predictors that influence vaccination of children. The other objective was to determine coverage of measles vaccination of the Measles Immunization Days (MID) 2005 for children aged 9 month to 6 years in a region of Umraniye, Istanbul, Turkey.

**Methods:**

A '30 × 7' cluster sampling design was used as the sampling method. Thirty streets were selected at random from study area. Survey data were collected by a questionnaire which was applied face to face to parents of 221 children. A Chi-square test and logistic regression was used for the statistical analyses. Content analysis method was used to evaluate the open-ended questions.

**Results:**

The complete vaccination rate for study population was 84.5% and 3.2% of all children were totally non-vaccinated. The siblings of non-vaccinated children were also non-vaccinated. Reasons for non-vaccination were as follows: being in the village and couldn't reach to health care services; having no knowledge about vaccination; the father of child didn't allow vaccination; intercurrent illness of child during vaccination time; missed opportunities like not to shave off a vial for only one child. In logistic regression analysis, paternal and maternal levels of education and immigration time of both parents to Istanbul were found to influence whether children were completely vaccinated or non-vaccinated. Measles vaccination coverage during MID was 79.3%.

**Conclusion:**

Efforts to increase vaccination coverage should take reasons for non-vaccination into account.

## Background

High vaccination coverage is important in control and elimination of vaccine preventable diseases in a country. In Turkey the Expanded Program of Immunization (EPI) aims for 95% coverage for each antigen and complete vaccination schedules for 90% of children under 1 year of age [1]. All the vaccines included in the national vaccination schedule (Bacille Calmette Guerin (BCG), Oral Polio Vaccine, Diphtheria-Pertussis-Tetanus (DPT) vaccine, Measles and hepatitis B vaccine are provided free of charge in the primary health services all over Turkey. Although the coverage of all vaccines in our country is increased especially in the last ten years, the EPI targets has not been achieved yet [2] and there are still regional differences. In 2003, full vaccination (one dose of BCG, one dose of measles and three doses of DPT and OPV) coverage rate in the Istanbul region for children under 2 years was 62.3% [3].

Our research area is located in the Umraniye region of Istanbul, which is the biggest metropolitan city of Turkey. The population of the research area is nearly 72 000. The region is heterogeneous. Although most parts are rural, both urban and rural settlements are present. The region still receives a high rate of immigration from less developed parts of the country and primary level health care services are provided through only one health care center which is active since 2001. This health center provides ambulatory care, immunizations, reproductive health services and health education. There is one government hospital and many private outpatient clinics in the study area. The ratio of illiterate parents in the study population is higher than the Umraniye and Istanbul population [4]. According to a study in Umraniye region full vaccination rates for children less than 5 years and less than 1 year was 68.3% and 79.5%, respectively [4]. The Umraniye Health District reported in 2002, the full vaccination rate in our study area as 53.8%, which indicates that there is still a big gap between EPI targets and vaccination coverage rates in our study area.

To achieve the EPI targets, information about reasons for non-vaccination is very important. Since the routine data in local regions does not provide accurate information, vaccination coverage is estimated through surveys. We need community-based information about vaccination status and reasons for non-vaccination in order to increase vaccination coverage rates and implement interventions for control and elimination of vaccine preventable diseases in high-risk areas like our study area. Sociodemographic and socio-economic factors can be important determining factors for vaccination coverage rates. Community-based information about the vaccination coverage and about the reasons for non-vaccination can guide the midlevel health managers to determine the priorities in their localities and plan interventions improving the vaccination coverage and executing their plans with the limited sources.

The primary objective of the study was to determine the full vaccination coverage and reasons for non-vaccination and the effects of sociodemographic factors that influence the vaccination of children in order to establish strategies for the health center area. The other objective was to determine the measles vaccination coverage for children aged 9 month-6 years during the mass measles campaign-2005.

## Methods

A '30 × 7' cluster sampling was used as the sampling method. The '30 × 7' cluster sampling method is recommended by WHO as a rapid, simplified and economic sampling method in assessment of vaccination coverage [5]. Clusters were defined as streets. Map of the health center area was used to select the streets at random and thirty streets were selected in the region. Seven households from 30 clusters were sampled. The starting point was selected as the first or lowest household number for each street and then continued to the next nearest household until seven eligible children was obtained. Data were collected in a two-week period after the mass measles campaign. In each street, door-to-door visits and face-to-face interviews were conducted with parents who had children 9 month-6 years of age. 91.4% of the interviews were conducted with the mothers of the children. The target sample size was 210. However, since all the individuals of appropriate age living in the households falling into the sample were included; our sample size became 221. After informed consent the parents of children participated in a structured interview. The structured questionnaire used for data collection included items on relevant attributes of parents (e.g. educational level, employment, family income, insurance coverage, immigration status); whether there are non-vaccinated children other than the child in our sample in the household, and if the answer was yes, the reason for non-vaccination of those children; relevant attributes of children aged 9 month – 6 years (age, sex, number of siblings, birth order, whether the child is born at home or at a hospital, child's full vaccination history and the health facility where the routine vaccination services were taken). The vaccination histories of these children were assessed from their mother's recall (44.3%) and if available the vaccination cards (55.7%) were checked. Children who are less than 18 months old are accepted as completely vaccinated if they have had one dose of BCG, three doses of hepatitis B, OPV and DPT and one dose of Measles vaccine. Children above this age are accepted as completely vaccinated if the have had their booster doses for OPV and DPT vaccines additionally. The study was approved by the Marmara University Faculty of Medicine Research Ethics Committee.

### Statistics

Analyses were conducted by SPSS12.0 and Epi info 5.0 software. Chi-square test was used for the statistical analyses of categorical data and content analysis method was used to evaluate the open-ended questions about non-vaccination reasons. Characteristics that were found, through bivariate analyses, to be significantly associated with vaccination coverage were entered into a multivariate logistic regression model, to rule out the confounding factors and to determine which characteristics were independent predictors of vaccination status of the child. Two-sided significance tests were used and p < 0.05 was accepted as the level of statistical significance.

## Results

In total, data pertaining to 221 children were collected. The numbers of male and female participants were approximately equal (52.9% and 47.1% respectively). There was no statistically significant difference between the distribution of sex and age groups.

One fifth of the participants were the only child of their families, 38% of them had one sibling, 23.1% had two siblings and 19% had more than three siblings. Education, immigration time to Istanbul from another district part of the country and the employment status of both parents are presented in Table 1. 62.4% of employed fathers are working as construction workers who get daily pay and are uninsured; housepainters who are uninsured also; or as insured slop workers who earn a bare subsistence. Mean monthly income of households was 534 ± 272.8 YTL (381$ ± 194.3$).

The class of health insurance of the study population was as follows: Social Security Institution (49.3%), Green card (8.2%), Social Security Organization for the Artisans and self employed (6.8%) and Social Security Organization for the civil servant (1.8%). More than one third of the families (33.9%) do not have any health insurance.

37.5% of mothers and 30.8% of fathers were immigrated to Istanbul for at least twenty years ago from different districts of the country. 61.5% of the children were born at a state hospital, where 33.1% at a private hospital and 5.4% (12 children) at home. Four of the children who were born at home were born after his/her family has migrated to Istanbul.

The healthcare facility used for routine vaccination service was primary health care centers (84.1%), maternal and child health and family planning clinics (11.7%), private doctors or private hospitals (4.2%).

Measles vaccination coverage during MID-2005 was 79.3% in our study population. According to parents' recall and if available to their vaccination card (55.7%), full vaccination rate was 84.5% for all of the children and seven children (3.2%) were never been vaccinated before the mass measles campaign. The rest were under vaccinated. The six of seven children who were never been vaccinated were vaccinated with measles vaccine during the campaign. In one case father of the child didn't permit vaccination. There was no statistically significant difference between vaccination status of the child and the source of information of vaccination status (parents' recall/vaccination card).

We asked, "*Are there any children in the household, except the child in our study population, who are totally unvaccinated?*" and if the answer was "yes" the reason for being totally unvaccinated were asked. In sixteen of the households the answer was "*yes*". Below are the reasons for non-vaccination of children in these households listed with mothers self explanations. In table 2 the reasons are represented as groups.

Being at the village and having no knowledge about vaccination:

• *The one who is unvaccinated was born in the village. We couldn't know where to go for vaccination. After we migrated to Istanbul the health center was too far to us." *(an illiterate mother with six children).

• *"We were at the village as the other children were born. There was no vaccine at the village and we didn't have any knowledge. We were clueless at that time" *(an illiterate mother with seven children).

• *"No vaccine was fetched to our village; they were grown up in the village" *(an illiterate mother with six children).

• *"We were at the village. We didn't know vaccine at that time." *(primary school educated mother with three children).

• *"The child was born in Van. I lived with my mother-in-law and therefore couldn't go. They said that vaccines were applied at the school" *(an illiterate mother with three children).

• *"All of the other children are totally unvaccinated. My mother-in-law didn't allow us to go. The access was hard from the village and we lived in straightened circumstances" *(an illiterate mother with eight children).

• *We came from Erzurum nearly, we don't know vaccine, and we supposed that it costs Money. We do not know Turkish well*. (an illiterate mother with three children).

The father of child didn't allow vaccination:

• *"My husband said 'although I have never been vaccinated, you can see that there is no problem with me. I grow up', when I asked money for vaccination of child he didn't gave and said the child will get ill if vaccinated" *(primary school educated mother with four children).

• "*All of our children are totally unvaccinated. My husband doesn't permit vaccination. He doesn't allow us to go anywhere" *(an illiterate mother with four children).

• *"My husband doesn't give permission to vaccinate our biggest child; he says the child will get disabled if vaccinated. I vaccinated the other two children without telling him"*, (primary school educated mother with three children).

Illness of child and misinformation about the side effects of vaccine during illness or contraindications:

• *"They became ill very frequent, and I'm afraid that the vaccines make them more ill" *(primary school educated mother with three children).

• *"Child is ill, she has had earache very frequently and I was afraid of it, so I didn't want her to be injected" *(an illiterate mother with two children).

• *"The child was ill during infancy, we were afraid. Later she gets well but I didn't desire to vaccinate the child*" (primary school educated mother with four children).

Missed Opportunities:

• *"I have had taken my child for vaccination when we were living in village, the nurse said that there was no opened vaccine vial and she hadn't opened a new vial. She told me to come later again, but I couldn't go over again" *(primary school educated mother with four children).

• *"The nurse in the primary health care center sends us to the Tuberculosis Control Dispensary, and the nurse working at the Tuberculosis Control Dispensary said that we have to go to the primary health care center in our district. I have gotten nervous, and didn't toke him*" (primary school educated mother with two children).

• "*When the child was small, our family was in an economic distress, Later I thought that no vaccination is needed because the child was grown up. No body in the health center told me that vaccination is necessary for children over the age five." *(primary school educated mother with three children).

In bivariate analysis there was no statistical association with full vaccination rate and gender and age of child, paternal employment status, use of primary health center or other centers for routine childhood immunizations and presence of health insurance. Vaccination rate was higher in children with higher educated parents, higher family income, parents living in Istanbul for at least twenty years, who were born at hospital, had no or only one sibling than the children with less educated parents, low family income, parents living in Istanbul for less than twenty years, who were born at home, had more than one siblings and (Table 3).

A multivariate logistic regression model with variables that were significantly associated with vaccination coverage in the bivariate analysis included educational level of mother and father, number of siblings, birth order, and place of birth, monthly family income, and immigration time to Istanbul. From this model, the only 3 significant predictors of vaccination of a child were educational level of father and mother and immigration time of parents to Istanbul (Table 4).

## Discussion

Although specialized software is recommended to analyze data in which complex sample methods such as cluster sampling technique is used for sampling [6], we couldn't use it because of software limitations.

There is only one governmental health care center responsible for primary health care services in our study area. Because the records of the health center about the vaccination coverage were insufficient in our study area, we used the 30 × 7 cluster sampling method as a rapid and economic method in assessment of vaccination coverage.

Information about vaccination status of children taken from mothers' recall (44.3%) may be another limitation of our study. Only children who live in our study area and have been vaccinated regularly at the health care center are recorded in the health care centers' records. Because the phone number and home address of the child is not taken during these records we couldn't check the reliability of our data. However, studies reporting that information about vaccination status of children taken from mothers' recall is accurate are available [7-9]. According to the Turkish National Demographic Health Survey 2003 vaccination card availability is reported as 54% [10]. Studies from other parts of Turkey report that availability of the vaccination cards may be as low as 20% [11]. Absence of vaccination cards is a problem in developed countries also. A study reports that only one-third of the children had vaccination cards in the USA [12]. The evidence of no statistically significant difference between vaccination status of the child and the source of information of vaccination status (parents' recall/vaccination card) in our study may diminish the effect of this limitation.

As shown in Table 4, being full vaccinated for children whose mothers' educational level is at least primary school graduation is nearly nine times more than for children whose mother had no education. According to the results of Turkish Demographic Health Survey 2003 the ratio of women educated less than primary school is 21.8% in Turkey. Regional inequalities still present. Although in our study area ratio of women educated less than primary school seems to be lower than this ratio (Table 1), it is similar to less developed parts of the country [10]. A recent study from eastern part of Turkey reports that every level of education that the mother had above illiteracy increased the probability of a child being vaccinated [13]. There are many other studies from Turkey and other countries reporting that education of mother increases the vaccination chance of a child [14-19] and is an important predictor for childhood health conditions [20,21]. In our study population nearly all (six of seven) of the mothers who explain the reason for non-vaccination of their children are illiterate. It seems to be difficult to improve vaccination coverage without taken into account women's education. More educated the mothers more care for their children's health.

We find that the being non-vaccinated for children whose father had education less than secondary school (less than 8 years) were nearly 2.3 times more than the children whose father's educational level was more. There are other studies conducted in Turkey reporting that higher paternal education has positive affect on childhood health conditions. [22-25].

Internal immigration from less developed parts to more developed parts of the country, which is a distinctive feature of social life of less developed or developing countries, is one of the realities of our study area. Internal immigration has many negative effects on vaccination services and other health services [26]. In our study, children whose both parents are living in Istanbul at least for twenty years are vaccinated 3.4 times more than those whose one or more parent is living in Istanbul less than this time (Table 4). Mothers, who are not aware of vaccination before they have been immigrated to Istanbul, didn't vaccinate their children after they have been immigrated to Istanbul. This finding in our study connotes us that these mothers have not been properly informed about utilizing the primary health care facilities. A widely held opinion about vaccination and intercurrent illness, among mothers was another reason for non-vaccination. Misinformations about the side effects of vaccine during illness or false contraindications are reported as non-vaccination reasons in another study [27].

As there is an association between paternal education and maternal education and child health conditions informing about importance of vaccination and motivating mothers and fathers to request vaccination for their children seem to be important in regions which are less developed regions like our research area.

It is not possible to draw comprehensive conclusions about answers including fathers' comparison of their own childhood with their children ("*although I have never been vaccinated, you can see that there is no problem with me. I grow up"*) without qualitative researches. Because the data in this survey were collected by a questionnaire with one open-ended question, this does not clarify the problem that fathers do not permit mothers to vaccinate their children.

For control or elimination of vaccine preventable diseases, it is mandatory to increase the number of vaccinated people in the community. We believe that most of these reasons for non-vaccination can be overcame by informing the general public about the importance of vaccination. Among reasons for non-vaccination missed opportunities are the ones in which the solutions are most known. Studies about missed opportunities reporting implementation programs are available [28-30].

This study indicates that the full vaccination coverage in this area is low according to EPI targets of Turkey. Survey data were collected with a short questionnaire structured for collecting data for two main purposes: 1) to get information about the vaccination rate during the mass measles campaign and 2) to get information about totally non-vaccinated children in the households who were not in our sample and the reasons for their non-vaccinations. The non-vaccination status of children living in the households and reasons their non-vaccination were asked only by two questions (*are there any other children who are non-vaccinated in the household and if the answer was yes, what were the reasons for non-vaccination of those children*). This question was not asked for each separate vaccine and only general vaccination status was asked. This was another limitation of our study. However we believe that the results of our study can aid in explaining the reasons for non-vaccination of children in areas like Umraniye to the health care facilitators and can help to increase the vaccination coverage rates.

## Conclusion

The vaccination coverage in our study area may not be termed as very low. Nevertheless, there are groups where the vaccination level is relatively low and the reasons for their non-vaccination can be overcomed by more effort. If their vaccination levels are to be improved, the reasons for non-vaccination in these groups must be taken into account.

## Competing interests

The author(s) declare that they have no competing interests.

## Authors' contributions

SDT designed the study, reviewed the literature, collected data and and performed the statistical analysis, directed the project and were responsible for writing the paper. NB reviewed the paper. Both authors read and approved the final manuscript.

## Pre-publication history

The pre-publication history for this paper can be accessed here:



## Figures and Tables

**Table 1:** Education level and employment status of parents

	Mothers		Fathers	
	(N = 221)	%	(N = 221)	%
**Education status**				
Illiterate	37	16.7	10	4.5
Graduated Primary school	151	68.3	142	64.3
Graduated Secondary school	10	4.5	38	17.2
Graduated High school	21	9.5	29	13.1
Graduated University	2	0.9	2	0.9
**Employment status**				
Employed	6	2.7	210	95.0

**Table 2:** Reasons for non-vaccination of children in the households

**Grouped Reasons for non-vaccination (n = 16)**	**Number**	**% (95% CI)**
Being at the village and having no knowledge about vaccination	7	43.6 (0.23–0.69)
The father of child didn't allow vaccination	3	18.8 (0.007–0.19)
Illness of child and misinformation about the side effects of vaccines	3	18.8 (0.007–0.19)
Missed opportunity	3	18.8 (0.007–0.19)

**Table 3:** Relation of some characteristics with vaccination status of children

**C'haractersitics**	**Vaccination Status**		p value
		
	**Full vaccinated**	**Not full vaccinated**	
	**(n,%)**	**(n,%)**	
**Sex**			
Male	96 (82.1)	21 (17.9)	>0.05
Female	91 (87.5)	13 (12.5)	

**Education of mother**			
Illiterate	15 (48.5)	16 (51.5)	0.000
Graduated Primary school	141(89.8)	16 (10.2)	
Graduated Secondary school or higher education	31 (93.3)	2 (6.7)	

**Education of father**			
Illiterate	5 (50.0)	5 (50.0)	0.003
Graduated Primary school	63 (83.8)	6 (16.2)	
Graduated Secondary school or higher education	119 (91.3)	23 (8.7)	

**Place of birth**			
Home	6 (50.0)	6 (50.0)	0.004
Hospital	181 (86.6)	28 (13.4)	

**Health center used for routine vaccination**			
Primary health care center/maternal and child health and family planning clinics	179 (87.3)	26 (12.7)	>0.05
Private doctors/hospitals	8 (88.9)	1 (11.1)	

**Number of siblings**			
No	42 (95.5)	2 (4.5)	0.035
1	72 (85.7)	12 (14.3)	
2+	73 (78.5)	20 (21.5)	

**Birth order**			
First or second child	128 (88.3)	17 (11.7)	0.031
Third or later born child	59 (77.6)	17 (22.4)	

**Health insurance**			
None	59 (78.7)	16 (21.3)	>0.05
Green Card	13 (72.2)	5 (27.8)	
Other Social Securities *	115 (89.8)	13 (10.2)	

**Immigrated to Istanbul**			
At least one parent <20 years ago	123 (80.4)	30 (19.6)	0.005
Both parents ≥20 years ago	64 (94.1)	4 (5.9)	

**Monthly family income**			
<450 YTL	65 (73.9)	23 (26.1)	0.000
≥450 YTL	121 (91.7)	11 (8.3)	

**Total**	187 (84.5)	34 (15.5)	

* Social Security Institution, Social Security Organization for the Artisans and self employed or Social Security Organization for the civil servant

**Table 4:** Variables significantly associated with full vaccination of children (backward logistic regression – final model)

	**β**	**p**	**OR**	**(95% CI)**
**Education of mother**				
No education*				
Primary school	2,157	0,000	8,648	(3,520–21.244)
Secondary school or higher	2,496	0,000	12,131	(2,385–61.711)
**Education of father**				
Less than secondary school*				
Secondary school or higher	1.841	0.035	2.320	(1.28–6.501)
**Parents' living time in Istanbul**				
At least one parent is living in Istanbul since < 20 years*				
Both parents are living in Istanbul since ≥20 years	1,230	0.037	3.421	(1,077–10.865)

*reference category
